# Characteristics of astigmatism in Black South African high school children

**DOI:** 10.4314/ahs.v17i4.25

**Published:** 2017-12

**Authors:** Samuel Otabor Wajuihian

**Affiliations:** Discipline of Optometry, School of Health Sciences , University of KwaZulu-Natal, Private Bag X54001, Durban 4000, South Africa

**Keywords:** Astigmatism prevalence, school children, South Africa

## Abstract

**Background:**

Astigmatism impairs vision at various distances and causes symptoms of asthenopia which negatively impacts reading efficiency.

**Objective:**

The aim of conducting this study was to determine the prevalence and distribution of astigmatism and its relationship to gender, age, school grade levels and spherical ametropia.

**Methods:**

Using a multi-stage random cluster sampling, 1589 children who included 635 (40%), males, and 954 (60%), females were selected from 13 out of a sample frame of 60 schools. Their ages ranged between 13 and 18 years with a mean of 15.81±1.56 years. The parameters evaluated included visual acuity using the LogMAR chart and refractive errors measured using an autorefractor and then refined subjectively. Axis of astigmatism was presented in the vector method where positive values of J0 indicated with-the-rule, negative values described against-the-rule and J45 represented oblique astigmatism.

**Results:**

The mean cylinder power was −0.09 ± 0.27 and mainly with-the-rule, J0 = 0.01 ± 0.11. The overall prevalence of clinically significant astigmatism (≤ − 0.75 cylinder) in the sample was 3.1% [(95% Confidence interval = 2.1–4.1%)]. Cylinder of at least − 0.25 power was considered to classify astigmatism types. Thus, the estimated distributions of types of astigmatism were: axis- 11.5%, sphero-astigmatism 10.1% and magnitude-astigmatism 11.2% while 67.2% had no cylinder of any magnitude.

**Conclusion:**

The prevalence of astigmatism is relatively low in this population studied. Older children and those in high school grade levels were more likely to have with-the-rule or against-the-rule astigmatism. The prevalence of astigmatism were comparable within but not across regions.

## Introduction

Uncorrected refractive error including astigmatism is a common vision anomaly in school-aged children,^1^–^2^ causes visual impairment (VI) and is the second leading cause of treatable blindness worldwide.^1^–^2^ Astigmatism occurs when incident light rays from infinity do not converge to a single focal point on the retina due to an irregular curvature of the cornea (corneal astigmatism, obtained from keratometer) or of the lens surfaces.^3^–^6^ Refractive astigmatism (that is, total astigmatism) includes both corneal and internal astigmatism and is the type of astigmatism obtained through retinoscopy or subjective refraction.^3^ Refractive astigmatism may further be classified as sphero-cylinderical (defined in relation to association with spherical component), magnitude of astigmatism (defined according to magnitude or amount) and axis orientation^3^–^4^ (defined based on cylinder axis). Astigmatism is clinically important given its associated symptoms which include asthenopia, blurry vision at various viewing distances, spatial distortion in size, shape or inclination of objects and thus degrades image quality.^3^–^4^ Furthermore, astigmatism alters emmetropization and is associated with myopia progression and development of amblyopia.^5^–^7^ Therefore, knowledge of the distribution of astigmatism in this population will guide clinical practice, research and epidemiology in records and resource allocation.

The prevalence of astigmatism is influenced by factors such as race, ethnicity, gender and age.^5^–^7^–^9^–^10^ Studies have found racial variations in the distribution of astigmatism^8^–^48^ and the prevalence of astigmatism could be as high as 13.7 % in the Black,^14^ 49% in Asian^35^ and 33.3% in the Caucasia^38^ populations. Especially in the Caucasian and Asian populations, other characteristics of astigmatism appear consistent and these include that human infants have a high prevalence of clinically significant astigmatism, most of which is against-the-rule.^6^,^8^ School-aged children show a lower prevalence of astigmatism, which changes minimally during school years^3^,^8^,^11^,^32^,^39^ with an axis shift towards with-the-rule.^5^,^8^ Similarly, the ethnic variations of astigmatism have been well-documented.^9^–^10^,^32^,^40^ The Native American tribes including the Navajo, Zuni, Siouz, Tohono O'Odham and Zuni^10^ and Asians^32^ have been reported to have a relatively higher prevalence and magnitude of astigmatism.^9^,^32^,^40^

The prevalence and degree of with-the-rule astigmatism was found to be higher in American Indians than Whites,^9^–^10^ children of East Asian background had significantly higher proportions of oblique refractive astigmatism^32^ while astigmatism of more than 0.5 D was found to be more common in Whites than in Blacks.^6^ In general, most studies^8^–^51^ reported on astigmatism broadly, without details on the types and participants age range was not specific on high school populations. Although the types of astigmatism are not mutually exclusive, classifying astigmatism is relevant in understanding the relation of astigmatism to unaided visual acuity, visual perception and symptoms.^3^ The high school population is of interest given that they represent a period of relatively higher near task demands and a period in growth and development towards early adulthood which may influence the distribution of astigmatism. In addition, given the reported race and ethnic variations of the prevalence of astigmatism,^8^–^51^ it was considered of interest to study the characteristics of astigmatism in a population of children of Zulu ethnicity in South Africa. The aim of this study was to determine the prevalence of astigmatism, and its types and investigate their associations with age, gender, grade levels and spherical ametropia.

## Methods

### Study design

The study design was cross-sectional and the protocol was approved by the Biomedical Research Ethics Committee (ethical clearance reference number BE 177/12) of the University of KwaZulu-Natal, South Africa. Written informed consent for access to the schools was obtained from the Department of Education and the school principals and the study was conducted in accordance with the Declaration of Helsinki regarding research on human subjects.

### Study setting and sampling strategy

The target population was Black high school students in the rural areas of the uMhlathuze municipality which is within the District of Northern KwaZulu-Natal province. The participants for this study comprised 1,589 children; 635 males and 954 females whose ages ranged between 13 and 18 years and were South Africans of the Zulu ethnic group. The 13 participating schools were selected from a total sample frame of 60 high schools in the municipality using simple random sampling while the participants were selected using stratified multistage cluster random sampling where each school was considered a cluster. The baseline sample size was determined using the formula for a (descriptive) prevalence study:^52^

n = Zα^2^ × p (1-p) / d^2^, where: n = sample size,

Zα = 1.96= (CI: 95%) (level of significance), p=expected prevalence or proportion (in decimal point) =estimate of 17.9% prevalence estimate from our pilot study.

D = Precision or margin of error.

$$\text{n}=\frac{\text{(1.96)2 X (0.179 X 0.821)}}{\text{(0.03)2}}=627$$

with a design effect of 1.8 = (627 × 1.8) = 1129 and additional 460 participants were added to the number to compensate for non-response, missing data, sub-group analysis, and to enhance generalization of findings through larger sample size which gave a total sample number of 1,589. The demographics are shown in Table 1.

**Table 1:** Participants' demographics

Category	Frequency	Percent	Mean age	Median age
**Gender**				
Females	954	60	15.72±1.584	16.00
Males	635	40	15.94±1.521	16.00
**Overall**	1589	100	15.81±1.56	16.00
**Age**				
13	144	9.1		
14	221	13.9		
15	310	19.5		
16	315	19.9		
17	315	19.9		
18	283	17.7		
**Total**	1589	100		
**Age groups**				
13–15	675	42.50		
16–18	914	57.50		
**Total**	1589	100		
**Grade level**				
8–10	1140	71.8		
11–12	449	28.2		
Total	1589	100		

The total number of students enrolled for the study per school was obtained by dividing the total sampling frame (from the 60 schools) by the tentative sample size to obtain the sample interval. The total number of students per school was then divided by the sample interval to obtain the total number of students studied per school. One to two classes were randomly selected from each school grade of 8–12. At the class level, the registers were used as the sampling frame from which individual students were selected using systematic sampling approach where a random starting point was selected and every n^th^ (from sample interval) student examined. Some minor modifications were made for cases such as inadequate number of students in a class where participants were selected from the next class.

Information sheets assent and consent forms written in the English language and the children's' indigenous language (isiZulu) were distributed to selected students in the identified schools and consent forms for parents were sent to them through their children. The leaflets contained information that explained the purpose of the study. Students who returned their approved parental consent and assent forms were enrolled for the study. For the entire study, students were excluded if they had ocular diseases, any systemic conditions or medications which might affect their refractive status.

### Procedures for eye examination

The school principals provided rooms at a school venue where the visual examinations were conducted. The purpose and procedure for every technique was carefully explained to each participant before starting the eye examination. Validated optometric instruments were used and techniques were applied as described.^52^ To eliminate inter-examiner variability which negatively impacts on validity, all tests were performed by an optometrist experienced in performing the techniques as described.^52^ All vision testing was performed in the mornings between 08:30 hours and 13:30 hours, over a period of one year between March 2013 and May 2014.Visual acuity (VA) was assessed for each eye using the Logarithm of Minimum Angle of Resolution (LogMAR) chart (Precision Vision, USA) at both distance and near. Refractive errors were assessed objectively using an autorefractor ((MRK-3100; Huvitz, Gunpo, Gyeonggi, South Korea) and refined subjectively to the best visual acuity with maximum convex lens monocularly and binocularly.^52^ Cycloplegia was not applied as the study entailed several near tests (also screened from accommodative-vergence anomalies), which precluded its use. A + 2.00 D fogging lens was used to screen for latent hyperopia.^52^ The traditional clinical representations of refraction using sphere, cylinder, and axis, are not adequate for quantitative analysis.^53^ Therefore, the transformations, J0 and J45 in the vector notation^34^,^49^,^51^,^55^ was used to analyze axis-astigmatism and for descriptive purposes only. Unlike the traditional notation, a major advantage of the vector method is that it enables the means of different cylinder axis to be summarised and added without losing values.^51^ In addition, the approach is useful in vision research and clinical practice,^51^ contact lenses fitting, as well as, in cataract surgery (as detailed by Motel et al).^50^ The conversion of the conventional sphere, cylinder and axis into vector transformation can be achieved using the formula:^34^,^50^–^51^,^55^ M = S + C/2

J0 = −1/2 C cos (2 α)

J 45 = −½ C sin (2 α)

Where M= spherical equivalent, S=sphere power, C = negative cylinder power and α = cylinder axis in degrees. J0 component may be considered a Jackson cross cylinder (JCC) with axes at 180° and 90°. Positive values of J0 indicate with-the-rule astigmatism while negative values indicate against-the-rule astigmatism. J45 refers to a cross-cylinder set at 45° and 135°, representing oblique astigmatism. A positive value for J45 denotes that the negative cylinder axis of the JCC is at 45°^34^–^50^–^51^,^55^ and is termed levo-oblique^55^ astigmatism. A negative value of J45 denotes that the negative cylinder axis of the JCC is at 135°^34^,^51^,^55^ and is referred to as dextro-oblique.^55^ The definitions and classification criteria^34^,^50^–^51^–^54^–^55^ are as indicated in Table 2. Axis astigmatism was classified as withthe rule, against-the- rule or oblique. Sphero-cylinderical astigmatism was classified as simple myopic, compound myopic and mixed astigmatism while magnitude astigmatism was classified as low, moderate and high.^54^ (Table 2)

**Table 2:** Diagnostic criteria for types of astigmatism

Astigmatism	Criteria
**Astigmatism (all)**	At least −0.75 in minus cylinder notation
a) Magnitude-astigmatism	• Low astigmatism: 0.25 to 0.50 D • Moderate astigmatism: 0.75 to 2 D • High astigmatism: greater than 2.00 D **With-the-rule astigmatism (WTR): produced when the corneal** curvature is steepest in the vertical meridian. A positive J0 indicates WTR astigmatism. **Against-the-rule astigmatism (ATR):** produced when the steepest corneal meridian is horizontal. A negative J0 indicates ATR astigmatism.
b) Axis- astigmatism	**Oblique: (OBLQ):** Oblique astigmatism occurs when the steepest curve of the cornea is not in the vertical or horizontal meridians but lies in an oblique position. The J45 refers to a cross-cylinder set at 45° and 135°, representing oblique astigmatism. The J45 component describes a JCC with its axes at 45° and 135°. A positive value for J_45_ denotes that the negative cylinder axis of the JCC is at 45° and is termed levo-oblique astigmatism. A negative value of J45 denotes that the negative cylinder axis of the JCC is at 135° and is referred to as dextro-oblique.
c) Sphero-astigmatism	Simple myopic astigmatism (SMA): • one ray comes into focus in front of the retina • one ray comes into focus on the retina (Plano/ ≥− 0.50 D). Compound myopic astigmatism (CMA): • both points of light come into focus in front of the retina (≥− 0.5 D / ≥− 0.5 D). Mixed astigmatism (MXA): • one ray comes into focus in front of the retina • one ray comes into focus behind the retina (+ 0.5 D / ≥− 0.5 D).

## Statistical analysis

All data were analyzed by a statistician using the Statistical Package for Social Sciences (SPSS) version 21. Descriptive statistics for visual acuity and refractive error variables were presented with means and standard deviation, and median. The Kolmogorov-Smirnov (K-S) normality test was used to determine normality of the data. The Pearson chi-squared test was applied to determine relationship between age, gender and astigmatism variables as well as association between the vector and traditional methods of representing astigmatism axis. The Pearson correlations test was applied to compare correlations between right and left eye spheres. Logistic regressions were used to examine odd ratios and multivariate associations including relationships between gender, age and astigmatism types. Distributions of variables were presented using tables and proportions and corresponding 95% confidence intervals were presented as estimates of the prevalence.

## Results

### Participants' demographics

Of the 1589 participants, three students were excluded, one was diabetic, one had glaucoma and the other had corneal scars due to trauma. All participants selected participated in the study which gave a response rate of 100%. Thus, data was analyzed for 1,586 students: females 954 (60.16%) and males were 632 (39.84%). Six hundred and seventy five children (42.55%) were in the 13–15 and 914 (57.62%) in the 16–18 age groups.

The descriptive statistics for the refractive error parameters are indicated in Table 3 with cylinder powers presented in minus notation while the descriptive statistics for the categories (gender, age and grade levels) are indicated in Table 4. All participants had normal near visual acuity of N4.5. There was a strong significant positive correlation between right and left eye spherical equivalent (r=0.881, p<0.001). Thus, even though anisometropia was defined from a difference between left and right eye spherical equivalent values, only the results for the right eye are reported.

**Table 3:** Descriptive statistics for visual acuity and refractive variables

	Mean	SD	Median	Minimum	Maximum	1^st^ quartile	3^rd^ quartile	Skew	Kurtosis
N=1586									
Distance visual acuity right eye	0.04	14	0.00	0.00	1	0.00	0.00	3.13	12.49
Right eye sphere power	−0.02	0.47	0.00	−10	4	0.00	0.00	−9.63	168.27
Right eye cylinder power	−0.09	0.27	0.00	−5.00	0	0.00	0.00	−6.60	75.12
RE J45	0.00	0.16	0.00	−3.00	5.00	0.00	0.00	13.70	550.11
REM	−0.05	0.51	0.00	−11.00	4.00	0.00	0.00	−9.46	155.34
RE J0	0.01	0.11	0.00	−1.00	2.00	0.00	0.00	5.83	128.36

**Table 4:** Descriptive statistics for the gender, age and grade levels

Clinical measures	All	Gender	Age group	Grade levels			
	N=1586	Females N= 954	Males N=632	13–15 N=675	16–18 N=911	8–10 N=1140	11–12 N=446
	Mean , SD	Mean, SD	Mean, SD	Mean, SD	Mean, SD	Mean, SD	Mean, SD
Distance visual acuity right eye	0.04 ± 0.14	0.04 ± 0.14	0.03 ± 0.15	0.04 ± 0.15	0.04 ± 0.14	0.04 ± 0.15	0.03 ± 0.13
Right eye sphere power	−0.02 ± 0.47	0.02 ± 0.51	0.01 ± 0.42	−0.03 ± 0.47	−0.02 ± 0.48	0.02 ± 0.52	0.01 ± 0.33
Right eye cylinder power	−0.09 ± 0.27	0.08 ± 0.26	0.09 ± 0.27	−0.09 ± 0.23	−0.09 ± 0.29	−0.09 ± 0.29	0.07 ± 0.21
J45	0.00 ± 0.16	0.00 ± 0.18	0.004 ± 0.12	−0.01 ± 0.14	0.01 ± 0.17	0.04 ± 0.18	0.01 ± 0.05
REM	−0.05 ± 0.51	−0.05 ± 0.54	−0.04 ± 0.46	−0.05 ± 0.51	−0.05 ± 0.51	0.05 ± 0.56	0.04 ± 0.34
J0	0.01± 0.11	0.01 ± 0.11	0.00 ± 0.10	0.01± 0.09	0.00 ±0.12	0.01 ± 0.11	0.00 ± 0.08

SD=standard deviation. There was no significant difference in the variables between the categories. The median values for all variables were essentially zero therefore was not indicated on table.

### Prevalence of astigmatism and types

The prevalence of clinically significant astigmatism was 3.1% while 1,536 (96.9%) had no clinically significant astigmatism. In addition, 501 (32.8%) participants had various types of astigmatism while 1,085 (67.2%) did not have any astigmatism. (Table 5) The differences in frequency numbers are due to clinically significant astigmatism being defined as at least −0.75 whereas the types of astigmatism were defined as at least −0.25 DC. The prevalence of axis-astigmatism was 11.5%, sphero-astigmatism was 10.1%, magnitude astigmatism 11.2%.

**Table 5:** Prevalence of astigmatism

Astigmatism types	frequency	Percentage	95% confidence interval	Upper limit
	n	%	Lower limit	Upper limit
**Clinically significant astigmatism** **(≤ 0.75)**	50	3.1	2.1	4.1
**Greater than −0.75**	1536	96.9		
**Total**	1586	100		
**TYPES (classified as at least −0.25 cylinder** **power)**				
**No astigmatism types**	1085	67.20		
**Astigmatism types**	501	32.80		
	1586	100		
**Axis-astigmatism**			**Lower limit**	**Upper** **limit**
▪ With-the-rule	92	5.82	4.74	7.02
▪ Against-the-rule	64	4.03	3.10	5.03
▪ Oblique	7	1.64	1.07	2.39
▪ **Total**	163	11.5	8.91	14.44
**Sphero-astigmatism**				
▪ Simple myopic	66	4.17	3.26	5.30
▪ Compound myopic	57	3.60	2.76	4.67
▪ Mixed	37	2.34	1.67	3.24
▪ **Total**	160	10.1	8.00	11.6
**Amount-astigmatism**				
▪ Low	128	8.08	6.81	9.56
▪ Moderate	49	3.08	1.10	2.43
▪ High	1	0.06	0.00	0.41
▪ **Total**	178	11.2	8.4	11.2

Axis of astigmatism was analyzed using both the conventional direction of the axis approach and the vector method. There was a highly significant association and dependency between the two methods of defining axis-astigmatism. (p < 0.01) Regarding astigmatism types and gender, although WTR was more frequent in females (3.41%) than in males (2. 27%), the difference was not significant while with-the-rule and against-the-rule were more prevalent in the older than younger age groups. Furthermore, anisometropia was found to be associated with axis-astigmatism (p=0.001) while myopia was associated with low astigmatism. (p=0.001)

The prevalence of myopia, hyperopia and anisometropia was estimated from spherical equivalent refraction values and were: myopia (≤−0.50) was 7.1% (95%CI= 5.9 - 8.5 %), hyperopia (≥ 0.50)4.5% (CI=3.5–5.6%), anisometropia (a difference of ≥ 0.75 mean spherical equivalent refraction), 2.7%, (95% CI=2.37–4.17), amblyopia 0.44% (CI-0.48–1.8%) and emmetropia (< 0.50) was 88.3% (CI=86.8–89.9).

## Discussion

In this study, astigmatism was analyzed and characterised according to types and in relation to gender, age, and its association with other refractive error in high school children of Zulu ethnic group. The prevalence of clinically significant astigmatism was 3.1%. For the types of astigmatism, with-the-rule, simple myopic and low astigmatism were most frequent. The overall prevalence of astigmatism is lower than findings in some African populations including those by Naidoo et al^12^ and Kuma et al.^14^ (Table 6) The differences in findings between this and other studies in African populations^12^,^14^ may be related to differences in age and techniques applied to evaluate refractive errors. For example, the authors^12^,^14^ studied younger children and reported retinoscopy findings using cycloplegia while findings from present study were derived from subjective refinements of refractive parameters without which may yield lower cylinder power acceptance. However, findings from the present study are comparable to the 3% prevalence estimates reported by Megbelayin et al^16^ in Nigeria (who also didn't apply cycloplegia) but higher than results from East African populations^19^–^21^ who had participants aged between 12 and 19 years as in present study.

**Table 6:** Distribution of astigmatism in school children across different regions

Authors/reference	Country of study	Ethnicity	Age (years)	Sample size	Criteria	Prevalence (%)
**Present study**	South Africa	Blacks	13–18	1586	≤ −0.75	3.1
Naidoo et al^12^	South Africa	Black	5–15	5599	≤ −0.75	9.2
**West Africa**						
Soler et al^17^	Equatorial Guinea	Blacks	6 − 16	425	≤−0.75	11.8
Jiménez^18^	Burkina Faso	Blacks	6–16	325	≤−0.75	11.7
Ovenseri & Assien^15^	Ghana	Blacks	11–18	604	≤−0.50	6.6
Kumat et al^14^	Ghana	Blacks	12–15	2435	≤−0.75	13.7
Megbelayin^16^	Nigeria	Blacks	9–21	1175	≤ −0.50	3.0
**East Africa**						
Wedner et al^19^	Tanzania	Blacks	14–19	2511	N/A	0.1
Muma et al^20^	Kenya	Blacks	12–15	1439	N/A	0.3
Njeru^21^	Kenya	Blacks	13–18	164	N/A	1.1
**North Africa**						
Chebil et al^23^	Tunisia	Caucasoid	6–14	6192	≤−0.75	6.67
El-Bayoumy et al^24^	Egypt	Caucasoid	7–15	5839		17.0
**Middle East**						
Fotouhi, et al^28^	Iran	Caucasoid	14–18	5721	≤ −0.75	18.7
Rezvan et al^58^	Iran	Caucasoid	6–16	2020	≤ −0.75	11.5
**Europe**						
Czepita et al^29^	Poland	White	6–18	5724	≤ −0.75	4.0
Abdi et a^30^	Sweden	White	6–16	216	≤ −0.75	11.6
Donoghue^11^	London	White	12–13	669	≤ 1.00	21.0
**East Asia**						
He et al^42^	China (urban)	Asian	5 − 15	5053	≤ −0.75	33.6
Congdon et al^33^	China (rural)	Asian	13–17	1900	≤−0.75	1.7
Shih e t al^32^	Taiwan	Asian	7–18	10878	≤ −0.75	49.0
**South East Asia**						
Quek et al^41^	Singapore	Asian	15–19	946	≤ −0.75	36.6
Ho et al^43^	Singapore	Asian	12–16	628	≤−1 .00	23.6
Goh et al^44^	Malaysia	Asian	7–15	4634	≤−0.75	15.7
Paudel te al^45^	Vietnam	Asian	12–15	2238	≤−0.75	0.75
**South Asia**						
Dandona et al^46^	India (rural)	Asian	7–15	4414	≤ −0.75	3.8
Murthy et al^47^	India (urban)	Asian	5–15	6447	≤ −0.75	7.0
Pokharel et al^48^	Nepal	Asian	5 − 15	5526	≤ −0.75	2.2
**Australia**						
Junghans^31^	Australia	Mixed	3 −12	2697	≤− 1.0	27.0
Robai et al^38^	Australia	White	12		> 1.00	33.3
Huyn^39^	Australia	Mixed	6 and 12	2367	≤ −0.75	13.6
**Americas**						
Kleistein^36^	USA	Mixed	5–17	2523	≤− 1.00	28.4
Maul et al^35^	Chile	Mixed	5–15	5,293	≤ −0.75	19.0

The findings from studies on West African populations ranged between 3 and 13.7% whereas those from East African populations had lower prevalence estimates of between 0.1 and 0.3%. The range of prevalence appears higher in populations of Caucasoid Africans (which include Tunisia and Egypt) with a range of between 6.67 and 17% which appears higher than those from Black African populations (Table 6). For non-African populations, the prevalence of astigmatism varied considerably across regions and tended to be higher in the regions with highest myopia prevalence and includes the South, South East and East Asian countries (Table 6). The prevalence of astigmatism from these South East and East Asian countries are higher than those from Africa, Middle Eastern, European populations and even South Asia.

Clinically, in patients with astigmatism, the unaided acuity, visual perception as well as presence of symptoms, which affects reading efficiency is dependent upon the type of astigmatism present.^3^ Similar to the present study, several reports suggested that low astigmatism is the most prevalent magnitude of astigmatism in school-aged children.^3^,^11^,^39^ On sphero-astigmatism, the highest prevalence was simple myopic astigmatism, which contrasts findings from studies^16^,^32^ that found a higher prevalence of compound myopic astigmatism. The higher frequency of simple myopic astigmatism (and not compound myopic astigmatism) in the present study may be related to the relatively lower prevalence of myopia, as well as, age differences across studies. Participants in these studies^16^,^32^ were younger than those in the present study. Classification of astigmatism according to combination with spheres is not common in the literature.

Axis-astigmatism is the most reported type of astigmatism. 8–47 Although one study^32^ found against-the-rule to be most frequent, the trend of higher prevalence of withthe-rule > against-the-rule > oblique in present study corroborates most reports^10^,^16^,^25^,^28^,^37^,^40^,^58^ and appears to be a consistent pattern of distribution. Even though there was no significant difference between the traditional method of classifying astigmatism and the vector method used in the present study, the variations in the criteria applied to define the axis orientation such as different axis may introduce differences in prevalence estimates of axis-astigmatism. Unlike gender, the prevalence of againstthe-rule and with-the-rule astigmatism was higher in the older than in the younger age groups. The distribution of astigmatism in relation to age is influenced by several factors which include the changes that the cornea undergoes with age,^5^ the type of astigmatism and the participants` age range.^5^,^7^,^11^ Findings from present study agree with previous report which found that, during school age, myopic astigmatism and low magnitude astigmatism tend to be predominant.^5^,^7^,^11^ Only myopia was significantly associated with low astigmatism, a finding which corroborates those by Shih et al^32^ in Taiwanese school children.

The possible explanations for some findings on astigmatism and its types in relation to age and gender may be proposed. The changes in astigmatism with age are influenced by factors such as the type of astigmatism^5^,^7^, ^11^ and the age range considered.^5^,^7^,^11^ It has been observed that during school age, myopic astigmatism tends to increase while hyperopic astigmatism decreases with age.^5^,^7^,^11^ Similarly, the prevalence of low astigmatism but not high astigmatism has also been reported to increase throughout childhood. Furthermore, the changes in astigmatism axis and magnitude with advancing age are related to the changes that the cornea undergoes with age.^5^,^7^,^11^ During childhood (between the ages of 4 and 18 years), the cornea flattens, and there is a subsequent reduction in the magnitude of astigmatism with small degrees of withthe-rule astigmatism being most common.^5^,^7^,^11^

Regarding astigmatism types and gender, although withthe-rule was more frequent in females (3.41%) than in males (2. 27%), the difference was not significant. Findings on the gender distribution of astigmatism are diverse. 8–47 Besides differences in core study designs which could account for differences in findings, Garcia et al^6^ proposed that females are more likely to have higher prevalence of axis-astigmatism than males.^6^ The authors^6^ found that females tended to have larger degrees of upward palpebral fissure slanting than males which suggests that astigmatism axis orientation is related to palpebral fissure slant.

Taken together, findings from this study and another in a South Africa population^12^ suggest that astigmatism is relatively low in school-aged children in South Africa. Furthermore, astigmatism is predominantly low magnitude, with-the-rule and simple myopic. The findings suggest that school children of the Zulu tribe may not be at high risk for astigmatism. This, however, does not remove the negative consequences of astigmatic errors which may affect reading efficiency and academic performance in affected children. The prevalence estimates from South Asia are relatively lower than those from other Asian countries. It has been suggested that regardless of the geographic location of India for example, genetic analysis of the origins of Indians revealed that Indians are not closely related to East Asians in terms of population genetics.^61^

In general, comparing findings across studies is complicated by diversity in study designs. The factors which could account for differences in prevalence estimates reported across studies include variations in sampling methods, criteria applied to define refractive variables, types of populations studied, (clinical or non-clinical samples and participants' age). Other factors include differences in measurement techniques,^36^,^56^ as well as, race and ethnicity. Specifically, the variations of patterns of astigmatism across ethnicity and race may be related to genetic and environment factors which include near work, education, socio-economic status, chemical exposure and nutrition. 7,11,25 Poor nutrition has been suggested to cause reduced pressures from the upper lid which flattens the cornea in the horizontal meridian and steepens the vertical meridian. Consequently, there is increased corneal rigidity which results in increased corneal astigmatism. In addition, the greater tightness of the Asian eyelids and narrower palpebral apertures may be a contributing factor. 59–60 More appropriate comparison of findings may be obtained with a multi-ethnic-/racial study.

Several measures taken to minimize bias include that only one examiner performed all procedures and as much as possible, similar standards were used for testing equipment, room setting and illuminations across schools. Furthermore, participants were selected from same population using random sampling strategy, the recruitment process was tightly followed and the appropriate statistical tests with appropriate assumption were applied. Refractive error was evaluated using objective technique and refined subjectively which gave a true indication of the participants' refractive status as they were consistent in their subjective responses.

The strength of this study includes a firm research design and eligibility criteria, a high response rate (at 100%) and appropriate measures were taken to minimise bias. Table 6 provides readers a summary of prevalence estimates for astigmatism from different regions while presenting axis-astigmatism in the vector method will enhance its application in practice and clinical research. Furthermore, presentation of detailed descriptive statistic (Tables 3 and 4) will hint on the mean normative range for these variables reported. The study may be considered to have a high internal and external validity and is likely to be representative, and findings could be extrapolated to the target population of high school children in the population studied. Findings will be relevant to clinicians and researchers in furthering an understanding of the distribution of astigmatism in school children. A limitation of the present study is that refractive error was determined without the use of cycloplegia although Fouti et al^27^ found cycloplegia not to influence the prevalence of astigmatism in high school children in Iran. The use of cycloplegia remains controversial^37^ especially in a study with the logistics of its use in a school setting being questionable and cycloplegia may not yield significantly different outcome.^31^

## Conclusion

This study characterized astigmatism in a population of Black South African school children of the Zulu, which was not reported previously. The prevalence of clinically significant astigmatism was low and low magnitude, with-the-rule and simple myopic astigmatism were the most frequent types of astigmatism. With-the-rule and against-the-rule astigmatism were more prevalent in the older than younger age groups. The prevalence of astigmatism was comparable within the African regions but differed across regions. Future studies in other racial and ethnic populations in South Africa are recommended so that comparative inferences can be drawn.
